# Physiological amyloid-beta clearance in the periphery and its therapeutic potential for Alzheimer’s disease

**DOI:** 10.1007/s00401-015-1477-1

**Published:** 2015-09-12

**Authors:** Yang Xiang, Xian-Le Bu, Yu-Hui Liu, Chi Zhu, Lin-Lin Shen, Shu-Sheng Jiao, Xiao-Yan Zhu, Brian Giunta, Jun Tan, Wei-Hong Song, Hua-Dong Zhou, Xin-Fu Zhou, Yan-Jiang Wang

**Affiliations:** Department of Neurology and Centre for Clinical Neuroscience, Daping Hospital, Third Military Medical University, 10 Changjiang Branch Road, Yuzhong District, Chongqing, China; Department of Laboratory Medicine, Southwest Hospital, Third Military Medical University, Chongqing, China; Neuroimmunology Laboratory, Department of Psychiatry and Behavioral Neurosciences, Morsani College of Medicine, University of South Florida, Tampa, FL USA; Rashid Laboratory for Developmental Neurobiology, Silver Child Development Center, Department of Psychiatry and Behavioral Neurosciences, Morsani College of Medicine, University of South Florida, Tampa, FL USA; Townsend Family Laboratories, Department of Psychiatry, The University of British Columbia, Vancouver, BC Canada; School of Pharmacy and Medical Sciences and Sansom Institute, University of South Australia, Adelaide, SA Australia

**Keywords:** Alzheimer’s disease, Parabiosis, Amyloid-beta, Clearance, Liver, Kidney, Periphery

## Abstract

Amyloid-beta (Aβ) plays a pivotal role in the pathogenesis of Alzheimer’s disease (AD). The physiological capacity of peripheral tissues and organs in clearing brain-derived Aβ and its therapeutic potential for AD remains largely unknown. Here, we measured blood Aβ levels in different locations of the circulation in humans and mice, and used a parabiosis model to investigate the effect of peripheral Aβ catabolism on AD pathogenesis. We found that blood Aβ levels in the inferior/posterior vena cava were lower than that in the superior vena cava in both humans and mice. In addition, injected ^125^I labeled Aβ40 was located mostly in the liver, kidney, gastrointestinal tract, and skin but very little in the brain; suggesting that Aβ derived from the brain can be cleared in the periphery. Parabiosis before and after Aβ deposition in the brain significantly reduced brain Aβ burden without alterations in the expression of amyloid precursor protein, Aβ generating and degrading enzymes, Aβ transport receptors, and AD-type pathologies including hyperphosphorylated tau, neuroinflammation, as well as neuronal degeneration and loss in the brains of parabiotic AD mice. Our study revealed that the peripheral system is potent in clearing brain Aβ and preventing AD pathogenesis. The present work suggests that peripheral Aβ clearance is a valid therapeutic approach for AD, and implies that deficits in the Aβ clearance in the periphery might also contribute to AD pathogenesis.

**Electronic supplementary material:**

The online version of this article (doi:10.1007/s00401-015-1477-1) contains supplementary material, which is available to authorized users.

## Introduction

Alzheimer’s disease (AD) is the most common form of dementia among the elderly. Senile plaques containing amyloid-beta protein (Aβ) in the brain are a pathological hallmark of AD and they play a pivotal role in AD pathogenesis. The steady-state level of Aβ in the brain is determined by the balance between Aβ production and its clearance [47]. In the brain, Aβ can be cleared via microglial phagocytosis and proteolytic degradation by enzymes such as neprilysin (NEP) and insulin-degrading enzyme (IDE) [47]. Transport of Aβ from the brain into the peripheral blood has been demonstrated in both animal models and humans [28, 36]. There are several potential pathways for the efflux of brain Aβ into the periphery. These include transport across the blood–brain barrier (BBB) mediated by low-density lipoprotein receptor-related peptide 1 (LRP1) [39], drainage from interstitial fluid (ISF) into cerebrospinal fluid (CSF) via perivascular [35] or glymphatic pathways [15], reabsorption from CSF into the venous blood via arachnoid villi [40] and blood–CSF barrier [32], or into the lymphatic system from the perivascular and perineural spaces [15, 34], and possibly via meningeal lymphatic vessels [15, 27] (see review in [41]). However, whether brain-derived Aβ is physiologically catabolized in the peripheral tissues and organs, and the therapeutic potential of this peripheral Aβ catabolism for AD, remains largely unknown. In the present study, we investigated the physiological catabolism of brain-derived Aβ in the periphery, and examined its therapeutic potential for AD using a model of parabiosis between transgenic AD mice and their wild-type littermates.

## Materials and methods

### Participants and blood sampling

**Fig. 1:**
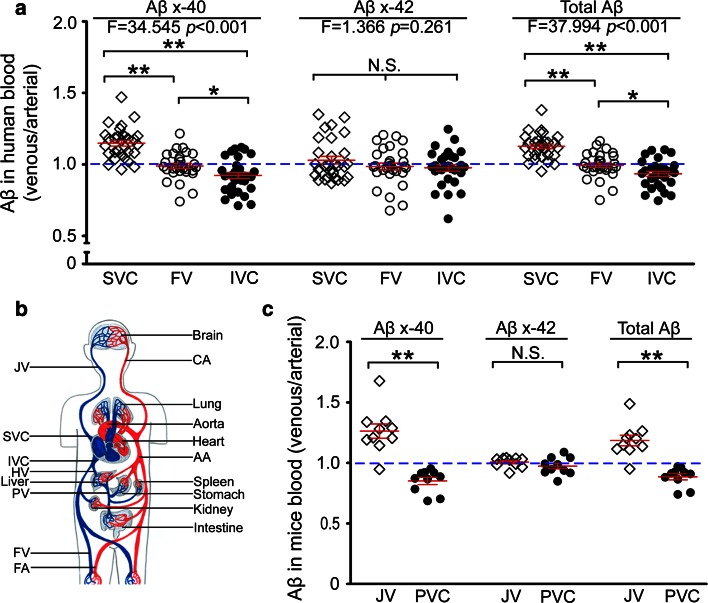
Aβ concentrations at different locations of the systemic circulation. **a** The venous/arterial (*V*/*A*) ratio of Aβ concentrations among blood samples from different venous locations in humans (*n* = 30). The *blue dotted line* represents Aβ concentration in the femoral artery (*FA*) as a reference. **b** A diagram of the circulation system and sampling locations for Aβ measurement. The superior vena cava (*SVC*) collects blood from the head containing brain-derived Aβ. The inferior vena cava (*IVC*) proximal to the hepatic vein (*HV*) collects blood from the lower part of the body including liver, kidney, and gastrointestinal tract. The femoral vein (*FV*) contains metabolites after the circulation through the lower limbs. The femoral artery (*FA*) contains blood virtually identical to that in the aorta and is used as a reference. **c** The venous/arterial (*V*/*A*) ratio of Aβ concentrations among blood samples from different locations in APPswe/PS1 mice (*n* = 10). The *blue dotted line* represents the Aβ concentration in abdominal aorta (*AA*) as a reference. *CA* carotid artery, *PV* portal vein, *JV* jugular vein, *SVC* superior vena cava, *IVC* inferior vena cava, *HV* hepatic vein, *FV* femoral vein, *FA* femoral artery, *PVC* posterior vena cava. Mean ± SEM, one-way ANOVA and Tukey’s test for human plasma and 2-tailed *t* test for mouse plasma, *P < 0.05, **P < 0.01. *N*.*S*. no statistical significance

The human study was approved by the Institutional Review Board of Daping hospital affiliated to Third Military Medical University. Informed consent was obtained from all individual participants included in the study. This study recruited 30 patients with atrioventricular reentrant tachycardia (AVRT) (left accessory pathways) who underwent radiofrequency catheter ablation (RFCA). Participants had an average age of 49.5 years (19–74 years) and included 17 males and 13 females, without cognitive impairment or dementia based on the clinical assessment. To measure the catabolism of Aβ in the periphery, we used time-matched arterial and venous peripheral blood samples as described previously [36]. Once the patients were deemed to undergo RFCA, catheters were placed in the left subclavian vein (SV), right femoral vein (FV), and right femoral artery (FA) simultaneously. Blood was collected from superior vena cava (SVC), inferior vena cava (IVC) proximal to hepatic vein (HV), the right FV and FA within 5 min (Fig. 1b). Five milliliters of blood were collected in vacuum blood tubes with anticoagulants at each site from the catheters. Blood was placed on ice immediately after sampling, and plasma was separated within 2 h and stored in polypropylene eppendorf tubes at −80 °C for further use. To avoid the influence of diurnal changes of Aβ levels, all the blood sampling were performed between 8:30 and 9:30 in the morning.

In a parallel animal study, the APPswe/PS1dE9 mouse model of AD (AD mice, Jackson Laboratories, Bar Harbor, MA) was used. This mouse model harbors the human amyloid precursor protein (APP) gent containing Swedish mutant and human presenilin 1 (PS1) gene encoding the deleted exon 9 mutation under control of mouse PrP promoter which directs the transgene expression predominantly in brain neurons [18], and develops amyloid plaques at 6 months of age [17]. Blood was sampled from the jugular vein (JV), abdominal aorta (AA), and posterior vena cava (PVC) of female APPswe/PS1dE9 mice aged 6 months (*n* = 10) following approval by the Third Military Medical University Animal Welfare Committee. Blood was sampled within 5 min at each site. The sampling was performed during 8:30–9:30 in the morning. Blood samples were examined for Aβ40 and Aβ42 levels following our protocols as described previously [3].

### Analysis for biodistribution of ^125^I-Aβ1-40

The radiolabelling of Aβ1-40 was performed following previous protocols [8]. Synthetic Aβ1-40 was iodinated with Na^125^I (specific activity 37 MBq/mg, China Institute of Atomic Energy) using the Iodogen technique (Pierce). Radioiodinated Aβ1-40 was separated from free iodine using a size exclusion column (Sephadex G-25, Pharmacia). The specific activity of radioiodinated Aβ1-40 was 1.065 MBq/μg. The radiochemical purity was >95 % trichloroacetic acid-precipitable.

Three-month-old male C57BL/6J mice received a bolus injection with 6.9 MBq ^125^I-Aβ in 0.3 ml of lactate Ringer’s solution (6 g/L NaCl, 0.2 g/L CaCl_2_, 3.1 g/L sodium lactate, 0.3 g/L KCl, pH 7.1) via the tail vein (*n* = 8). Mice were sacrificed by overdose of ketamine at 120 min after injection. The brain, skin, gastrointestinal tract, lung, heart, liver, spleen, kidney, and carcass were collected and weighted. The radioactivity (CPM values) of samples was measured with a wipe test counter (CAPRAC), and the resulting counts/min was normalized per gram of tissue. The radioactivity values are shown as % of total radioactivity or counts/min/gram of tissue.

### Parabiosis

APPswe/PS1dE9 transgenic (Tg) mice were bred in the animal facility of Daping hospital. All mouse husbandry procedures were approved by the Third Military Medical University Animal Welfare Committee. To exclude the influence of gender on brain Aβ deposition, female Tg mice were used in the present study. Each pair of mice was placed together in a cage for 1 month to allow the mice to adapt to each other [10, 11]. Female Tg mice and their age- and weight-matched female wild-type (Wt) littermates were selected for parabiosis including the parabiosis from 3-months of age to 9-months of age (as the group before Aβ deposition in the brain) and the parabiosis from 9-months of age to 12-months of age (as the group after Aβ deposition in the brain) (*n* = 8 per group). The age-matched female Wt (*n* = 8) and Tg mice (*n* = 8) without parabiosis were used in parallel as controls. The parabiosis was performed following procedures as previously described [31]. Briefly, animals were anesthetized with ketamine (100 mg/kg), xylazine (20 mg/kg), and acepromazine (3 mg/kg), and placed in a parallel orientation. A left lateral incision was made on one mouse while a right one was made on the partner mouse, extending from the base of the ear toward the hip. The incision included skin and muscle along thorax and abdomen. The opposing muscle layers of the two mice were joined with 5-0 silk sutures. The scapulae of the mice were fixed together with 4-0 silk sutures. The corresponding dorsal and ventral skin was sutured with 4-0 silk. After the surgery, the parabiotic mice were allowed to recover in a warm and clean environment before being transferred into the husbandry area. Prophylactic antibiotic treatment (enrofloxacin, 5 mg/kg) was started 1 day prior to the surgery and continued for 1 week. All animals received analgesic/anti-inflammatory treatment (acetylsalicylic acid 5 mg/kg) for 2 weeks.

### Brain sampling

Animals were euthanized by overdosing with pentobarbital (0.08 g/kg). Blood was sampled from the right atrium of the heart, followed by intracardial perfusion with 100 ml of 0.1 % NaNO_2_ in phosphate buffer. Brains were sampled and weighed. Left hemispheres were fixed in 4 % paraformaldehyde for histological analysis, and right hemispheres were snap frozen in liquid nitrogen and stored at −80 °C for biochemical analysis.

### Histology and quantification

Coronal sections of the brain were cut at a 35 μm thickness with a cryosectioning microtome and stored at 4 °C in PBS containing 0.1 % sodium azide until use. For the histology analysis, a series of five equally spaced tissue sections (~1.3 mm apart) spanning the entire brain were used for each type of staining.

#### Aβ plaques

The compact Aβ plaques were visualized with Congo red staining following our previous protocols [51]. In brief, sections were treated with working sodium chloride solution (containing sodium chloride saturated in 80 % alcohol and 0.01 % sodium hydroxide) at room temperature (RT) for 20 min, then placed directly into working Congo red solution (containing saturated Congo red in working sodium solution) for 1 h, and then dehydrated rapidly in absolute alcohol. The total Aβ plaques containing both compact and diffuse plaques were visualized with antibody 6E10 immunohistochemistry.

#### Cerebral amyloid angiopathy (CAA)

Cerebral amyloid angiography (CAA) were visualized with Congo red staining and quantified following previously described protocols [48]. In brief, sections were stained with Congo red and images were collected at the selected regions from frontal cortex to hippocampus of each mouse brain under the same illumination conditions. Quantification of Congo red staining was performed using the Image J software. A series of standard images representing extremes of high and low levels of Congo red staining were used to establish segmentation threshold using the RGB method to distinguish positively stained pixels. CAA was manually selected from each image, and the measurement of positively stained pixels was performed under the same RGB segmentation values. This yields the amyloid load due to Congo red staining of blood vessels. Meanwhile, the average number of CAA profiles per section was calculated by dividing the total count of CAA with the total number of sections as in our previous study [50].

#### Microhemorrhage

Sections were stained for hemosiderin with 2 % potassium ferrocyanide in 2 % hydrochloric acid for 15 min, followed by a counterstaining with 1 % Neutral Red solution for 10 min at room temperature [51]. Microhemorrhage profiles were counted under microscopy, and the average number of hemosiderin deposits per section was calculated.

#### Neuronal degeneration

Apoptosis of neuronal cells was detected with NeuN and Caspase-3 double immunofluorescence staining. Neuronal loss and neurite degeneration were detected with NeuN and microtubule-associated protein (MAP)-2 double immunofluorescence staining.

#### Astrocytosis and microgliosis

Immunohistochemistry was used to visualize astrocytosis and microgliosis with anti-CD45 antibody to detect activated microglia and anti-glial fibrillary acidic protein (GFAP) antibody to detect astrocytes.

Quantification was conducted by an investigator who was blinded to the group information of the samples. The area fraction and/or density of positive staining was quantified with ImageJ software.

### ELISA assays

Frozen brain was homogenized in liquid nitrogen and successively extracted with TBS, 2 % SDS, and 70 % formic acid solutions following our previous protocols [51]. Levels of Aβ40 and Aβ42 were measured using ELISA kits (Covance). Concentrations of inflammatory cytokines IL-6, IL-1β and TNF-α in brain extracts and blood were quantitatively measured with ELISA according to the manufacturer’s instructions (eBioscience).

### Western blotting

The levels of molecules or enzymes involving Aβ metabolism, phosphorylated Tau, and synapse-related proteins were analyzed using Western blotting. Proteins in the animal brain homogenate were extracted with RIPA buffer. Samples were loaded on SDS-PAGE (4–10 % acrylamide) gels. Separated proteins were transferred to nitrocellulose membranes. The blots were probed with the following antibodies: anti-APP C-Terminal (171610, Millipore) which recognizes full-length APP (APPfl) and C-terminal fragment (CTF)-β, anti-BACE1 (Millipore), anti-NEP (Millipore), anti-receptor for advanced glycosylation products (RAGE, Millipore), anti-LRP-1 (5A6, Calbiochem), anti-IDE (Epitomics), anti-phosphorylated-Tau antibodies including anti-pS396 (Signalway) and anti-pS199 (epitomics), anti-Synaptophysin (Millipore), anti-Synapsin-1 (Millipore), anti-PSD95 (Millipore), anti-PSD93 (Millipore) and anti-β-actin (Sigma-Aldrich). The membranes were incubated with IRDye 800CW secondary antibodies (Li-COR) and scanned using the Odyssey fluorescent scanner. The band density was normalized to β-actin for analysis.

### Statistical analysis

All data represent the mean ± SEM. Statistical analysis included 2-tailed Student’s *t* test for the comparison of two groups, one-way ANOVA and Tukey’s test for the comparison of multiple groups when required. Normality and equal-variance testing was performed for all assays. *P* < 0.05 was considered significant. All analyses were completed with SPSS software, version 10.0.

## Results

### Aβ levels in different locations of the circulation

A recent study in humans demonstrates the efflux of Aβ from the brain to the plasma [36]. However, the fate of brain-derived Aβ in the periphery remains unclear. To examine the catabolism of brain-derived Aβ in the periphery, we measured Aβ levels in the different parts of the circulation including: (a) the superior vena cava which collects blood from the head containing brain-derived Aβ, (b) the inferior vena cava proximal to the hepatic vein which collects blood from the lower part of the body including liver, kidney and gastrointestinal tract, (c) the femoral vein which contains metabolites after the circulation through the lower limbs, and (d) the femoral artery which contains blood virtually identical to that in the aorta and used as a reference (Fig. 1b, Supplemental Table 1). In addition, Aβ levels in blood from the jugular vein, the abdominal aorta and the posterior vena cava were examined in AD mice aged 6 months (Supplemental Table 2). The Aβ concentrations in different locations of the circulation were divided by time-matched arterial Aβ concentrations to generate the venous/arterial (V/A) ratio. We found that V/A ratios of Aβ40 and total Aβ from the superior vena cava were significantly higher than that from the inferior vena cava in humans (Fig. 1a), and V/A ratios of Aβ40 and total Aβ from the jugular vein of AD mice were also higher than that from the posterior vena cava (Fig. 1c). Interestingly, V/A ratios of Aβ40 and total Aβ from the inferior vena cava were significantly lower than that from the femoral vein in humans (Fig. 1a). These findings suggest that brain-derived Aβ in the arterial blood is physiologically cleared when it goes through the capillary beds of the peripheral organs and tissues, and that the internal organs remove significant amounts of arterial Aβ. There were no differences in Aβ42 concentrations among different locations of the circulation in both humans and AD mice (Fig. 1a, c), suggesting that Aβ42 is not a sensitive marker to reflect peripheral Aβ catabolism possibly due to its low level in blood [36].

### Distribution of ^125^I-Aβ1-40 in peripheral tissues and organs

We next investigated where Aβ is metabolized in the periphery in wild-type mice. Two hours after intravenous (i.v.) bolus injection of ^125^I labeled Aβ through the tail vein different organs and tissues were collected and weighed. In addition, radioactivities were detected respectively following a previous protocol [8]. We found that the injected radioactivity was located mostly in the liver, kidney, gastrointestinal tract, and skin while the rest was located in the carcass (Supplemental Fig. 1a). Moreover, by normalizing the data according to the mouse weights, we also observed significant amounts of radioactivity uptake in the liver, kidney, gastrointestinal tract, and skin but little in the carcass (Supplemental Fig. 1b). The radioactivity uptake in the brain accounted for only 1.67 % of total injected radioactivity, suggesting that peripheral organs and tissues are the main places of Aβ clearance in the periphery. This is particularly true for the liver and kidney, which are suggested to be the major organs for uptake of exogenous Aβ in the blood [8].

### Parabiosis reduces brain amyloid deposition

The above findings raise a critical question of whether the clearance of Aβ by the peripheral system has any impact on the pathogenesis of AD. We utilized isochronic parabiosis to test the efficacy of peripheral clearance of Aβ in alleviating the amyloid burden in the brains of APPswe/PS1dE9 transgenic mice. After parabiosis between transgenic and wild-type mice, the circulation of the parabionts was connected so that Aβ species in the blood of the parabiotic transgenic mice could be transported into the wild-type mice. Thus, the transgenic mice acquired an additional peripheral system from wild-type mice. This model provided a reliable approach to test the Aβ clearing capacity of the peripheral system.

Parabiosis was performed before Aβ deposition at 3 months of age and samples were collected for analysis after Aβ deposition at 9 months of age (Supplemental Fig. 2a). After parabiosis, the blood levels of both Aβ40 and Aβ42 of the parabiotic transgenic mice [pa(3-9mon)Tg] were significantly lower than that of the control transgenic mice (Supplemental Fig. 2b). Additionally, blood Aβ levels of the parabiotic wild-type mice [pa(3-9mon)Wt mice] were comparable to that of pa(3-9mon)Tg mice. These findings suggest that Aβ from the pa(3-9mon)Tg mice entered the circulation of the pa(3-9mon)Wt mice.

**Fig. 2:**
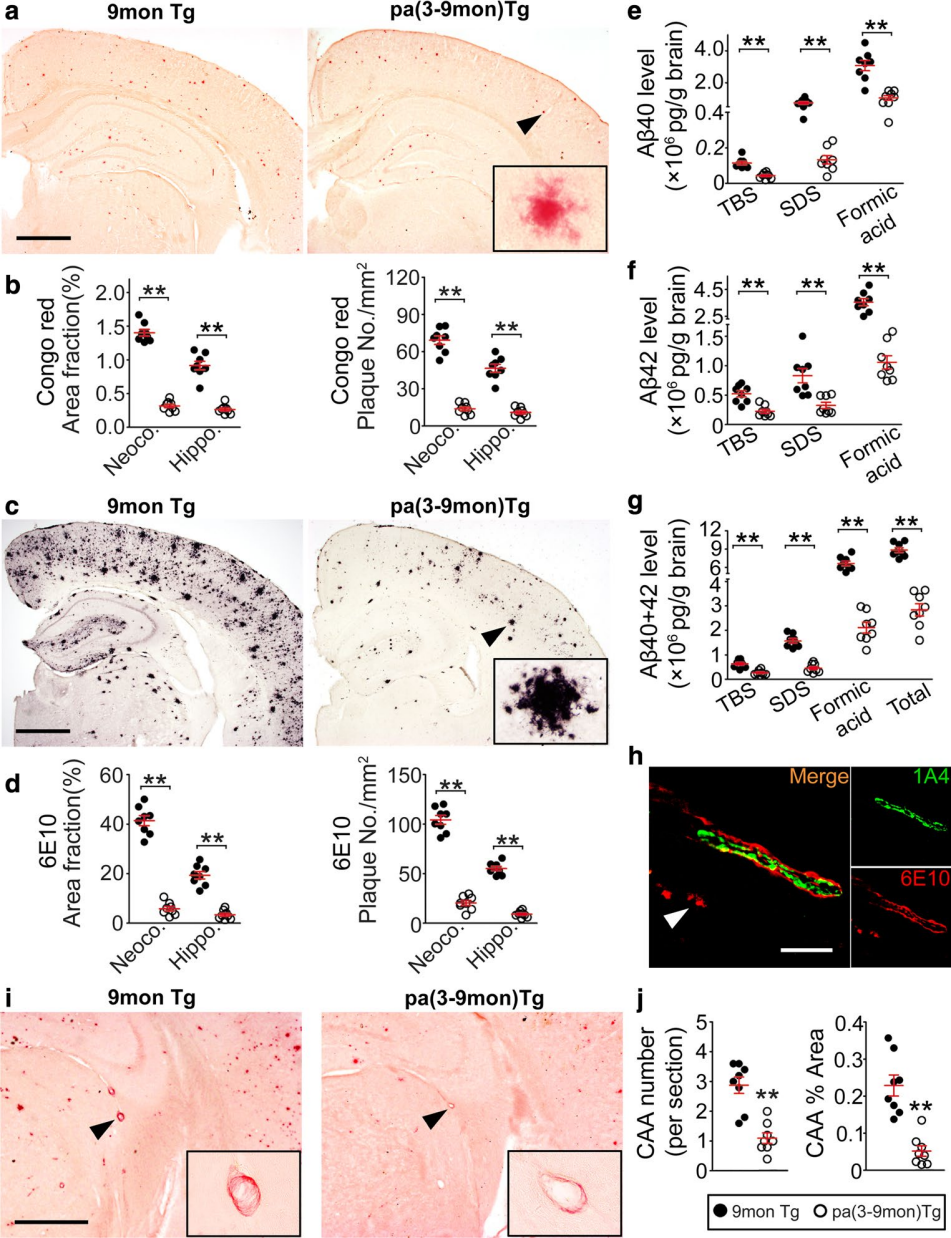
Parabiosis reduces brain amyloid burden of AD mice. **a**, **c** Representative images of Congo red and 6E10 immunohistochemical staining in neocortex and hippocampus in 9mon Tg and pa(3-9mon) Tg mice. *Insets* show the representative morphology at higher magnification. *Scale bars* 500 μm. **b**, **d** Comparison of the area fraction and density of Congo red or 6E10-positive Aβ plaques in the neocortex (*Neoco*.) and hippocampus (*Hippo*.) between 9mon Tg and pa(3-9mon) Tg mice. **e**, **f**, **g** Comparison of Aβ40, Aβ42 and total Aβ levels measured with ELISA in TBS, 2 % SDS and 70 % formic acid fractions of brain extracts between 9mon Tg and pa(3-9mon) Tg mice. **h** Illustration of Cerebral amyloid angiopathy (*CAA*) by immunofluorescence with the antibody to Aβ (*6E10*) and smooth muscle in the vessel wall (*1A4*). The *arrow* indicates the Aβ plaques in the brain parenchyma near the CAA. *Scale bars* 100 μm. **i** CAA visualized using Congo red staining. *Insets* show the representative morphology of CAA stained by Congo red at higher magnification. *Scale bars* 500 μm. **j** Comparison of numbers of CAA profiles and area fraction of CAA between 9mon Tg and pa(3-9mon) Tg mice. *n* = 8 per group, mean ± SEM, 2-tailed *t* test, *P < 0.05, **P < 0.01

Compared with control transgenic mice, pa(3-9mon)Tg mice had dramatic reductions in area fraction and plaque density of both compact plaques stained with Congo red, total plaques stained with 6E10 in neocortex and hippocampus (Fig. 2a–d), Aβ deposition in vessel walls (CAA) (Fig. 2h–j), and microbleed profiles (Supplemental Fig. 3). The total area fraction of amyloid deposition in both hippocampus and neocortex was reduced by 70 % for Congo red positive plaques, and 86 % for 6E10 positive plaques in pa(3-9mon)Tg mice relative to control transgenic mice (Fig. 2b, d). After parabiosis, the levels of Aβ40 and Aβ42 were significantly reduced in the brain homogenates of pa(3-9mon)Tg mice in comparison with control transgenic mice, with a reduction of total Aβ by 68 % (Fig. 2e–g). These data suggest that providing an additional peripheral system by parabiosis can substantially prevent Aβ deposition in the brain of transgenic mice.

We further investigated whether enhancement of peripheral Aβ clearing capacity could reduce Aβ burden after extensive Aβ deposition in the brain using the parabiosis models between 9-month-old female transgenic and age-matched female wild-type mice. Similarly, after 3 month parabiosis, the Congo red and 6E10-positive Aβ plaque burdens in the neocortex in pa(9-12mon)Tg mice were significantly lower than that of 12 months old transgenic control, and there was no significant increase in Aβ plaque burdens in pa(9-12mon)Tg mice when compared with 9-month-old transgenic mice (Supplemental Fig. 4). Taken together, our findings suggest that enhancing Aβ clearance in the periphery has both preventive and treatment potential for AD.

As the amyloid burden is associated with both Aβ production and clearance, we further investigated the metabolism of APP after parabiosis. There were no significant differences in the levels of full-length APP (APPfl), C-terminal fragment (CTF)-α, CTF-β, beta-secretase (BACE)-1, Aβ-degrading enzymes IDE and NEP, and Aβ transport receptors across BBB (LRP-1 and RAGE) in the brain between the pa(3-9mon)Tg mice and the control transgenic mice (Supplemental Fig. 5), suggesting that the reduction of brain Aβ burden after parabiosis was not due to changes in Aβ production or degradation. In addition, as the parabiotic mice were of the same age, the reduced brain Aβ burden was not due to the rejuvenation mechanism as observed in a previous parabiosis study [42].

### Parabiosis attenuates AD-type pathologies

**Fig. 3:**
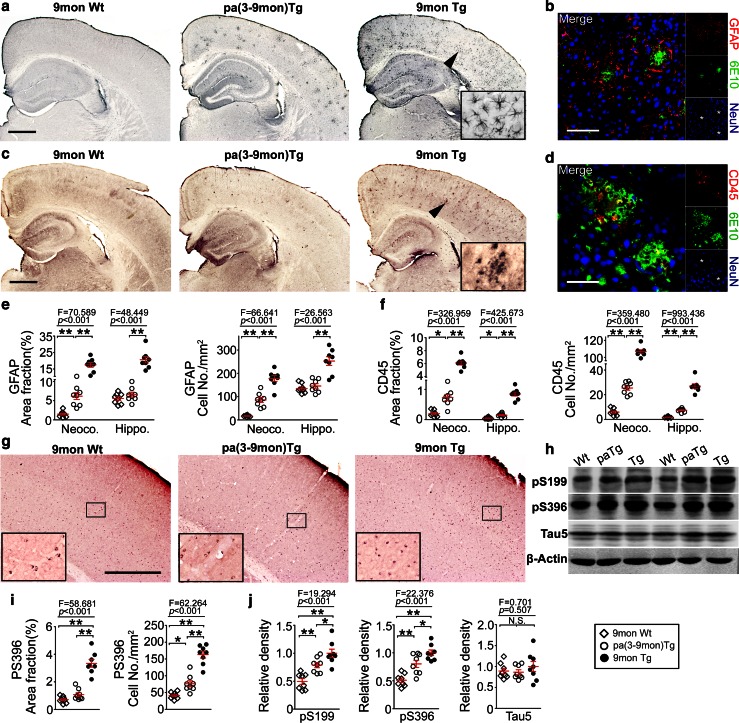
Parabiosis attenuates neuroinflammation and Tau phosphorylation. **a** Representative images of astrocytosis stained with anti-GFAP antibody in the brain. *Insets* show the representative morphology at higher magnification. *Scale bars* 500 μm. **b** Immunofluorescence image of amyloid deposition and astrocytosis co-stained with 6E10 (*green*) and anti-GFAP (*red*) antibodies. Aβ plaques were surrounded by activated astrocytes. *Scale bars* 100 μm. **c** Representative images of microgliosis stained with anti-CD45 antibody in the brain. *Insets* show the representative morphology at higher magnification. *Scale bars* 500 μm. **d** Immunofluorescence image of amyloid deposition and microgliosis co-stained with 6E10 and anti-CD45 antibodies. Aβ plaques were surrounded by activated microglia. *Scale bars* 50 μm. **e**, **f** Comparisons of area fraction and cell density of astrocytosis (**e**) and microgliosis (**f**) in the neocortex (*Neoco*.) and hippocampus (*Hippo*.) among pa(3-9mon)Tg mice, control Tg mice and Wt mice. **g** Representative images of intracellular Tau phosphorylation stained with anti-pSer396 antibody in the brain. *Insets* show the representative morphology at higher magnification. *Scale bars* 500 μm. **h** Western blot assays of phosphorylated Tau at multiple sites including pSer199, pSer396, and total Tau (*Tau5*) in the brain homogenates of parabiotic Tg mice (*PaTg*), control Tg mice and wild-type mice (*Wt*). **i** Comparisons of area fraction and cell density of cells containing phosphorylated Tau stained with anti-pSer396 antibody in the neocortex among parabiotic Tg mice (*PaTg*), control Tg mice and wild-type mice. **j** Comparison of band density for phosphorylated Tau (*pS199* and *pS396*) and total Tau (*Tau5*) shown in **h** among pa(3-9mon)Tg mice, control Tg mice and Wt mice. *n* = 8 per group, mean ± SEM., one-way ANOVA and Tukey’s test, *P < 0.05, **P < 0.01. *N*.*S*. no statistical significance

We investigated whether other AD pathologies could also be attenuated in the brain after parabiosis. In comparison with control transgenic mice, inflammation was significantly attenuated in the brain of pa(3-9mon)Tg mice, as reflected by decreased levels of astrocytosis and microgliosis (Fig. 3a–f), and reduced levels of proinflammatory cytokines including TNF-α, IL-1, and IL-6 (Supplemental Fig. 6). The levels of phosphorylated Tau (pS199 and pS396), but not total Tau (Tau5), were significantly reduced in the brain of pa(3-9mon)Tg mice after parabiosis (Fig. 3g–j).

**Fig. 4:**
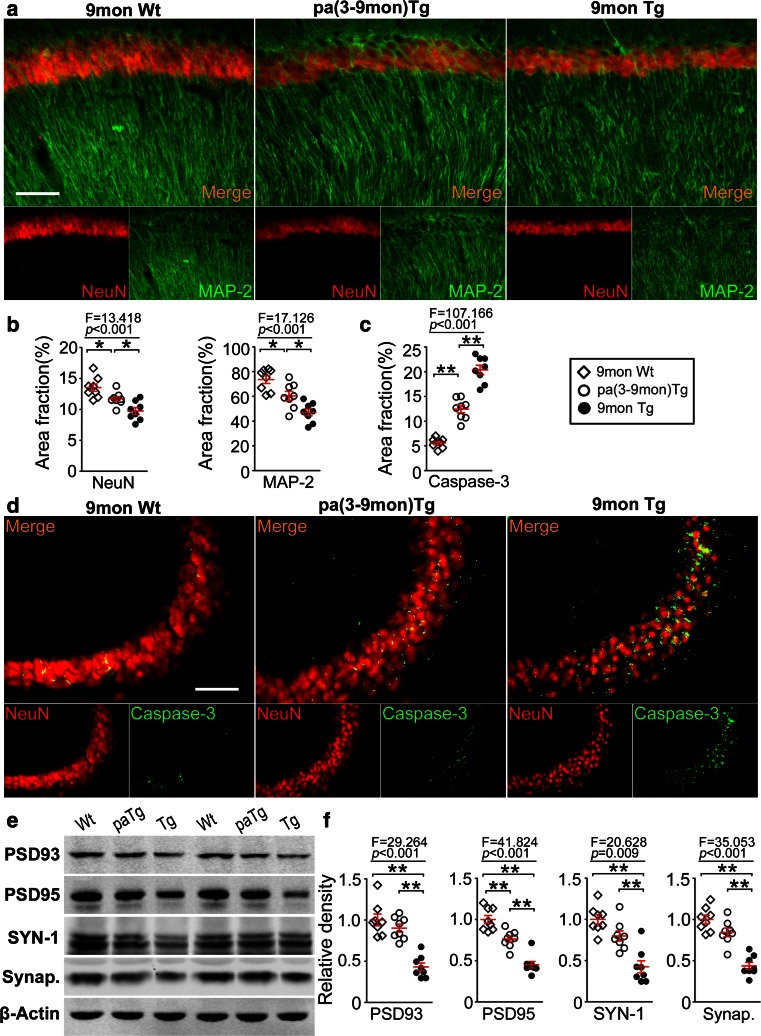
Parabiosis alleviates neuronal degeneration and loss in the hippocampus of pa(3-9mon)Tg mice. **a** Representative images of neurons and dendrites at CA1 region of hippocampus stained with anti-NeuN and anti-MAP-2 immunofluorescence in pa(3-9mon)Tg mice, control Tg mice, and wild-type (*Wt*) mice. *Scale bars* 100 μm. **b** Comparison of the area fractions of NeuN and MAP-2 staining among pa(3-9mon)Tg mice, control Tg mice, and Wt mice. **c** Comparison of area fractions of caspase-3 staining among pa(3-9mon)Tg mice, control Tg mice, and Wt mice. **d** Representative images of neuronal apoptosis at CA3 region of hippocampus as stained with activated caspase-3 immunofluorescence. *Scale bars* 100 μm. **e** Western blot assays of synapse-associated proteins including PSD93, PSD95, synapsin1 (*SYN-1*), and synaptophysin (*Synap*.) in brain homogenates of pa(3-9mon)Tg mice, control Tg mice, and wild-type (*Wt*) mice. **f** Comparisons of band density of PSD93, PSD95, SYN-1, and Synap. among pa(3-9mon)Tg mice, control Tg mice, and wild-type mice. *n* = 8 per group, mean ± SEM, one-way ANOVA and Tukey’s test, *P < 0.05, **P < 0.01

Compared with control transgenic mice, the pa(3-9mon)Tg mice displayed less neuronal apoptosis and damage as reflected by caspase-3 staining in hippocampus (Fig. 4a–d), and higher levels of synapse-associated proteins including PSD93, PSD95, synapsin-1, and synaptophysin in the brain (Fig. 4e, f). These data suggest that neurodegeneration was attenuated in the brains of transgenic mice after parabiosis.

## Discussion

In the present study, we provide evidence of physiological catabolism of brain-derived Aβ in the peripheral system in humans and mice. Using this model of parabiosis we, for the first time, revealed that the physiological ability of Aβ clearance in the periphery has a significant impact on preventing Aβ accumulation in the brain, as reflected by around 80 % reduction of brain Aβ deposition after parabiosis. Liver, kidney, gastrointestinal tract and skin are the major places for Aβ catabolism in the periphery. The other AD-associated pathologies, including tau phosphorylation, neuroinflammation, and neuronal degeneration, were also significantly attenuated in the brains of AD mice after parabiosis.

We also found that peripheral clearance of brain-derived Aβ exists physiologically. A recent study revealed the efflux of brain-derived Aβ to peripheral blood [36]. Consistently, we found that the blood Aβ levels in the superior vena cava were higher than that in the femoral artery, suggesting that Aβ effluxes from the brain into peripheral blood. However, the previous study did not address the question of whether brain-derived Aβ is catabolized in the peripheral system. This information is critical for understanding AD pathogenesis and development of approaches for prevention and treatment of AD. In the present study, we found that blood Aβ levels in the inferior vena cava were significantly lower than that in the femoral artery in human. This pattern of changes was confirmed in APPswe/PS1 mice whose blood Aβ levels in jugular vein were higher than that in abdominal aorta, and blood Aβ levels in posterior vena cava were lower than that in abdominal aorta. These findings suggest that brain-derived Aβ in the arterial blood is physiologically cleared when it goes through the capillary bed of the peripheral organs and tissues, in particular, liver, kidney, gastrointestinal tract, and skin.

The parabiosis model was used to test the efficacy of natural peripheral Aβ clearance in removing brain Aβ in AD mice. After parabiosis, the circulations of the parabiotic mice were connected, and thus Aβ species in the blood of the parabiotic AD mice could be transported to the wild-type mice for clearance. Parabiosis provides parabiotic AD mice an additional set of peripheral tissues and organs from wild-type mice. As such it is a reliable model to test the efficacy of peripheral Aβ clearance. After parabiosis, brain Aβ plaques of AD mice were dramatically reduced by around 80 %. It is unlikely that changes in Aβ production, degradation, or transport across BBB play dominant roles in the reduction of brain Aβ of the parabiotic AD mice, as there were no significant changes in the expression of APPfl, CTF-α, CTF-β, BACE1, IDE, NEP, LRP-1, and RAGE. A recent study shows that parabiosis between APPswe/PS1 mouse with ApoE gene and APPswe/PS1 mouse without ApoE gene did not reduce compact plaque burden in the brain of APPswe/PS1 mice with the ApoE gene [31]; suggesting that the systematic inflammation or immune responses induced by the parabiosis surgery would not be responsible for the large reduction of brain Aβ burden observed in the present study. In addition, both acute and chronic systematic inflammation are suggested to accelerate but not halt Aβ deposition and disease progress of AD [13, 14, 19]. Moreover, we found that human Aβ generated from the parabiotic AD mice can be detected in the blood of the parabiotic wild-type mice, indicating that Aβ from the AD mice entered the circulation of the parabiotic wild-type mice. These findings suggest that parabiosis reduces brain Aβ burden through the clearance by peripheral tissues and organs, rather than through modulation of brain Aβ production, degradation, receptor-mediated transport across the BBB, or systematic inflammation induced by the surgery.

The present study reveals the importance of natural Aβ catabolic capacity of the peripheral system in clearing brain Aβ. Based on the above findings, we can calculate the percentage of brain Aβ clearance by a singular peripheral system. We define the brain Aβ burden of an AD mouse as 100 % and the reduction of brain Aβ by adding an additional peripheral system as 68 % of the brain Aβ burden of the AD mice as indicated in our study. Accordingly, the total Aβ in the brain of the AD mice should be 100 % + 100 % × 68 %. Thus, a singular peripheral system can remove 40.4 % of Aβ burden in the brain as derived from the equation: clearance rate of a singular peripheral system = % reduction of brain Aβ in parabiotic AD mice/[100 % + % reduction of brain Aβ in parabiotic AD mice]. This calculation is close to the estimation that efflux of Aβ to peripheral blood accounts for 50 % of total brain Aβ clearance in humans [36], suggesting that the physiological Aβ clearance capacity of the peripheral system provides an important mechanism against Aβ accumulation in the brain.

Our findings also imply that dysfunction of peripheral Aβ clearance may contribute to the development of AD. Indeed, a previous study showed that moderate renal impairment is associated with the increased risk for developing dementia [38]. We also found that the levels of Aβ in blood are higher in patients with renal failure than normal controls [26]. Recent studies reveal that an allele (rs3865444^C^) of CD33, which attenuates the Aβ internalization capacity of monocytes, is associated with the increased risk of AD [2, 30]. These findings support the notion that peripheral Aβ metabolism is involved in AD pathogenesis.

Clearance of brain Aβ represents a promising anti-Aβ therapeutic strategy. Peripheral Aβ clearance is proposed to be a safer therapeutic approach for AD [25, 45] as adverse effects, such as neuroinflammation, vasogenic edema, and microhemorrhage occurred in trials of immunotherapies which target Aβ clearance likely due to the entry of anti-Aβ antibodies into the brain and cerebral vessel walls (see review in [24]). Recently, we found that an antibody against N-terminus of Aβ can facilitate the conversion of Aβ fibrils into more toxic Aβ oligomers, and then induce neuronal death in the brain [23]. This might be a reason for the acceleration of brain atrophy after clearance of Aβ by antibodies in the AN1792 trials [7]. In addition, the antibody against N-terminus of Aβ can cross bind to APP on the surface of neurons and promote the generation of Aβ [5, 20], and binding of autoantibodies to neurons is associated with increased level of intracellular Aβ [29]; suggesting that antibodies against Aβ or neuronal surface APP molecule may also increase Aβ production once they enter the brain. Taken together, these findings suggest that removal of brain Aβ from a peripheral approach might be a safe therapeutic way for AD.

Some efforts were made by previous investigations to test the therapeutic efficacy of peripheral Aβ clearance for AD. Enhancement of Aβ degradation in liver by *Withania somnifera* extracts significantly reduced Aβ levels in the brain [37]. Peripheral administration of a single chain antibody (scFv) to Aβ is as effective as intracranial administration of the scFv in reducing brain Aβ burden, but does not increase brain levels of soluble Aβ, which has potential to form more toxic oligomeric species [46]. However, intravenous infusion of antibody solanezumab as a peripheral “sink” inducer failed to remove brain Aβ deposits [6]. Also, peripheral administration of NEP reduces blood Aβ levels but fails to clear Aβ accumulated in the brain [12, 44]. In contrast, other studies indicate that continuous peripheral expression of NEP gene in skeletal muscle is able to reduce brain Aβ burden [9, 21, 22]. A critical reason for these conflicting results is that these Aβ clearing agents may also enter the brain, directly interact with Aβ, and even prohibit brain Aβ clearance under certain circumstances. For example, a monoclonal antibody 266, the parental antibody of solanezumab, can enter the brain and form the complex with soluble Aβ species; thus, retarding the efflux of Aβ from the brain into the blood [49]. In addition, NEP also catabolizes a variety of substrates other than Aβ; some of which (i.e. bradykinin, atrial natriuretic factor) are involved in brain Aβ accumulation or transport across BBB [16, 33]. Thus, these studies did not take a pure approach of clearing Aβ in the periphery. In this regard, reliable methodologies are needed to test the efficacy of peripheral Aβ clearance. Herein, by using a model of parabiosis to provide AD mice with an additional peripheral system, we provide a clear answer to the controversial issue that peripheral clearance of Aβ is a valid therapeutic approach for AD. Although the parabiosis cannot be applied to human for AD therapy, enhancement of Aβ catabolism in the periphery such as liver, kidney, or gastrointestinal tract would be a potential valid approach for developing AD therapy in the future.

The property of a therapeutic agent being able to penetrate the BBB is generally regarded as a prerequisite for anti-AD agents. Based on our findings drugs which directly act on Aβ in the periphery would have a therapeutic significance even though they do not pass through BBB. Indeed, a parabiosis study shows that circulating ApoE, which does not enter the brain, acts as a peripheral sink to induce net efflux of Aβ from the brain [31]. Thus, drug development against Aβ in the future can focus on the clearance of Aβ from the circulation and might be a promising therapeutic approach for AD.

Like other studies using the model of parabiosis [1, 4, 43], we did not perform behavioral tests to examine whether cognition is improved after parabiosis, as separation of the parabionts causes substantial lesions and stress to the animals. But consistent with the reduction in brain Aβ burden parabiosis also alleviated the other AD pathologies including tau phosphorylation, neuroinflammation, and neurodegeneration. This suggests that Aβ catabolism in the periphery is able to generate therapeutic efficacy.

In conclusion, our study reveals the substantial contribution of the peripheral system to the clearance of brain Aβ, providing proof-of-concept evidence that development of drugs and therapies for AD could be focused on peripheral rather than central Aβ clearance [25]. This study also implies that deficits in the Aβ clearance of the peripheral system might also contribute to AD pathogenesis.

## Electronic supplementary material

Supplementary material 1 (DOCX 14338 kb)
