# Supplementary material for: Physiological amyloid-beta clearance in the periphery and its therapeutic potential for Alzheimer’s disease
Source: Acta Neuropathol. 2015 Sep 12;130(4):487–99. doi: 10.1007/s00401-015-1477-1 (PMC4575389; doi:10.1007/s00401-015-1477-1)
Supplement: Supplementary file 1 — Supplementary material 1 (DOCX 14338 kb) [file 401_2015_1477_MOESM1_ESM.docx]

**Supplemental Information**

**Title: Physiological amyloid-beta clearance in the periphery and its therapeutic potential for Alzheimer’s disease**

Yang Xiang^1^, Xian-Le Bu ^1^, Yu-Hui Liu ^1^, Chi Zhu^1^, Lin-Lin Shen^1^, Shu-Sheng Jiao^1^, Xiao-Yan Zhu^2^, Brian Giunta^3^, Jun Tan^4^, Weihong Song^5^, Hua-Dong Zhou^1^, Xin-Fu Zhou^6^, Yan-Jiang Wang^1^

Yang Xiang, Xian-Le Bu and Yu-Hui Liu contributed equally to this work.

1. Department of Neurology and Centre for Clinical Neuroscience, Daping Hospital, Third Military Medical University, 10 Changjiang Branch Road, Yuzhong district, Chongqing, China.

2. Department of Laboratory Medicine, Southwest Hospital, Third Military Medical University, Chongqing, China.

3. Neuroimmunology Laboratory, Department of Psychiatry and Behavioral Neurosciences, Morsani College of Medicine, University of South Florida, Tampa, Florida, USA.

4. Rashid Laboratory for Developmental Neurobiology, Silver Child Development Center, Department of Psychiatry and Behavioral Neurosciences, Morsani College of Medicine, University of South Florida, Tampa, Florida, USA.

5. Townsend Family Laboratories, Department of Psychiatry, The University of British Columbia, Vancouver, British Columbia, Canada.

6. School of Pharmacy and Medical Sciences and Sansom Institute, University of South Australia, Adelaide, Australia.

Address correspondence to: Yan-Jiang Wang, Phone: +86 23 68757850; E-mail: yanjiang_wang@tmmu.edu.cn.

**Supplemental Table 1. Aβ levels of blood sampled from arteries and veins of patients**

| Patient | Sex | Age | Diagnosis | Aβ40 (pg/ml) | | | | Aβ42 (pg/ml) | | | |
| --- | --- | --- | --- | --- | --- | --- | --- | --- | --- | --- | --- |
|  |  |  |  | SVC | FA | FV | IVC | SVC | FA | FV | IVC |
| 1 | F | 19 | AVRT | 1827.61 | 1859.55 | 2072.72 | 1711.19 | 463.20 | 429.50 | 459.47 | 421.70 |
| 2 | M | 68 | AVRT | 2357.10 | 2180.55 | 2334.07 | 2300.06 | 419.23 | 452.29 | 510.46 | 511.88 |
| 3 | M | 33 | AVRT | 1991.97 | 1903.69 | 1881.09 | 1844.45 | 446.51 | 444.75 | 448.58 | 436.34 |
| 4 | F | 52 | AVRT | 2126.46 | 1596.81 | 1552.15 | 1419.69 | 364.82 | 409.92 | 404.20 | 398.03 |
| 5 | M | 31 | AVRT | 1813.49 | 1773.36 | 1769.88 | 1637.36 | 552.60 | 450.84 | 501.52 | 448.36 |
| 6 | M | 61 | AVRT | 2938.92 | 1999.99 | 1913.76 | 1613.04 | 416.18 | 431.10 | 411.11 | 462.58 |
| 7 | M | 68 | AVRT | 5982.00 | 5131.67 | 3802.65 | 3692.10 | 1353.82 | 1018.69 | 784.30 | 872.63 |
| 8 | F | 59 | AVRT | 4878.72 | 3907.85 | 3985.55 | 3034.13 | 850.97 | 717.27 | 511.66 | 569.36 |
| 9 | F | 65 | AVRT | 2116.35 | 1771.28 | 1775.97 | 1619.44 | 411.68 | 455.34 | 490.79 | 567.81 |
| 10 | M | 35 | AVRT | 2900.88 | 2403.33 | 2319.36 | 2179.55 | 449.56 | 506.86 | 519.42 | 455.73 |
| 11 | F | 71 | AVRT | 2286.96 | 2022.14 | 1897.85 | 1666.51 | 410.24 | 429.50 | 423.35 | 444.33 |
| 12 | M | 44 | AVRT | 2563.83 | 2138.50 | 1873.28 | 1891.96 | 393.55 | 432.55 | 415.72 | 415.51 |
| 13 | M | 50 | AVRT | 3540.95 | 3149.65 | 3101.94 | 2915.57 | 812.61 | 750.66 | 731.83 | 737.56 |
| 14 | M | 42 | AVRT | 1269.88 | 1157.53 | 1407.85 | 1281.60 | 405.58 | 409.92 | 404.20 | 405.57 |
| 15 | M | 45 | AVRT | 2266.90 | 1897.75 | 1810.75 | 1688.13 | 407.19 | 468.98 | 565.98 | 491.65 |
| 16 | M | 44 | AVRT | 1432.30 | 1319.95 | 1473.83 | 1463.56 | 414.73 | 420.51 | 491.84 | 444.59 |
| 17 | M | 50 | AVRT | 5143.70 | 4365.28 | 4118.78 | 3286.15 | 619.21 | 629.48 | 735.47 | 600.63 |
| 18 | M | 71 | AVRT | 6122.75 | 5183.83 | 4918.17 | 4322.53 | 1006.98 | 788.54 | 639.85 | 623.31 |
| 19 | F | 29 | AVRT | 3093.48 | 3101.50 | 3338.71 | 2936.34 | 488.88 | 462.88 | 437.44 | 457.00 |
| 20 | F | 58 | AVRT | 2862.68 | 2252.78 | 2188.13 | 1780.43 | 414.73 | 415.86 | 391.98 | 438.78 |
| 21 | F | 46 | AVRT | 1951.84 | 2024.07 | 2054.35 | 1937.38 | 396.60 | 443.14 | 413.80 | 437.99 |
| 22 | F | 48 | AVRT | 2888.84 | 2680.19 | 2625.97 | 2882.01 | 519.22 | 517.45 | 511.56 | 453.97 |
| 23 | M | 62 | AVRT | 2040.59 | 1576.30 | 1255.05 | 1496.35 | 404.51 | 419.03 | 357.20 | 394.74 |
| 24 | F | 74 | AVRT | 4894.66 | 4130.45 | 3554.84 | 3492.16 | 505.36 | 509.17 | 500.55 | 552.89 |
| 25 | F | 39 | AVRT | 3581.61 | 2965.49 | 2866.18 | 2110.57 | 579.22 | 635.54 | 647.73 | 747.12 |
| 26 | M | 38 | AVRT | 2611.07 | 2224.58 | 2391.78 | 2208.29 | 488.92 | 548.65 | 527.88 | 527.71 |
| 27 | F | 63 | AVRT | 2642.77 | 2318.80 | 2438.07 | 2133.09 | 572.47 | 481.80 | 576.80 | 524.86 |
| 28 | M | 21 | AVRT | 1729.30 | 1590.51 | 1550.52 | 1739.30 | 795.82 | 589.73 | 575.61 | 464.82 |
| 29 | M | 40 | AVRT | 1844.74 | 1778.67 | 1933.39 | 1991.86 | 451.37 | 479.78 | 476.62 | 475.88 |
| 30 | F | 60 | AVRT | 3091.78 | 2888.18 | 2997.41 | 3077.47 | 959.68 | 762.48 | 516.42 | 472.93 |

Abbreviations: F, female; M, male; AVRT, atrioventricular reentrant tachycardia; SVC, superior vena cava; FA, femoral artery; FV, femoral vein; IVC, interior vena cava.


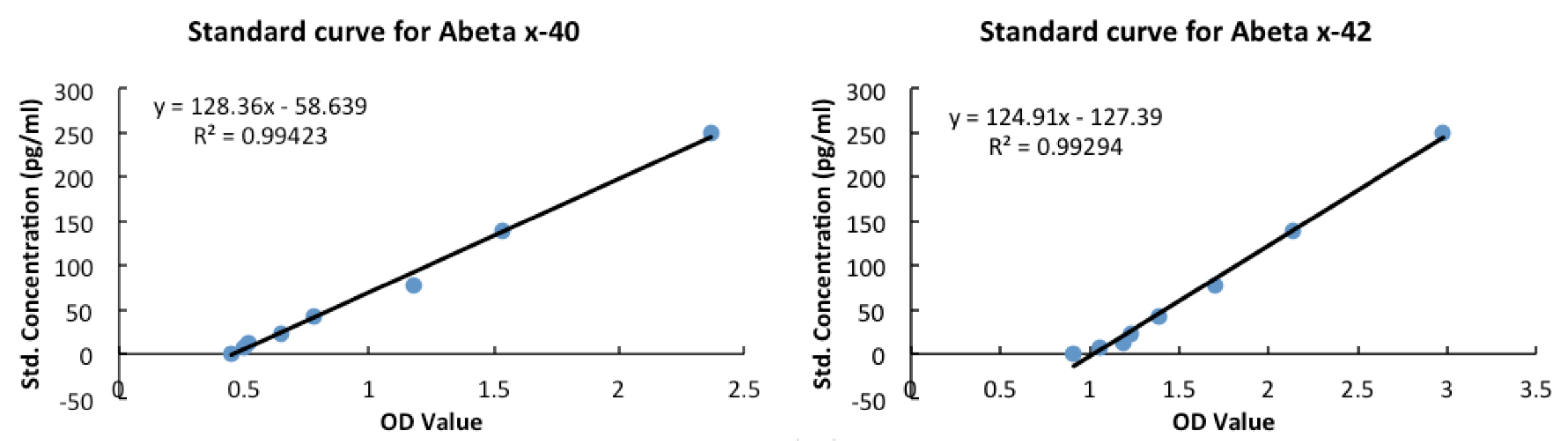


**Supplemental Table 2. Aβ levels of blood sampled from arteries and veins of APPswe/PS1dE9 transgenic mice**

| Mouse | Sex | Age(month) | Aβ40 (pg/ml) | | | Aβ42 (pg/ml) | | |
| --- | --- | --- | --- | --- | --- | --- | --- | --- |
|  |  |  | JV | AA | PVC | JV | AA | PVC |
| 1 | F | 6 | 4627.38 | 3458.72 | 3017.37 | 1864.73 | 1811.86 | 1774.22 |
| 2 | F | 6 | 5892.16 | 4874.09 | 4492.01 | 2019.32 | 2223.58 | 2403.80 |
| 3 | F | 6 | 6873.38 | 5368.30 | 4941.37 | 2527.15 | 2455.87 | 2283.65 |
| 4 | F | 6 | 4529.66 | 3960.95 | 2785.08 | 1530.71 | 1497.07 | 1257.49 |
| 5 | F | 6 | 4282.95 | 3324.15 | 2283.65 | 1212.71 | 1235.14 | 1160.65 |
| 6 | F | 6 | 6656.31 | 3968.15 | 3115.09 | 2024.90 | 1864.73 | 1932.01 |
| 7 | F | 6 | 4537.67 | 3766.30 | 3264.08 | 1572.36 | 1610.01 | 1474.64 |
| 8 | F | 6 | 4963.80 | 3691.01 | 3377.02 | 1263.18 | 1235.14 | 1123.00 |
| 9 | F | 6 | 3807.95 | 4021.02 | 3833.59 | 1879.15 | 1961.65 | 1932.01 |
| 10 | F | 6 | 7240.24 | 6079.59 | 5390.73 | 2206.76 | 2133.86 | 2208.36 |

Abbreviations: F, female; JV, jugular vein; AA, abdominal aorta; PVC, posterior vena cava.


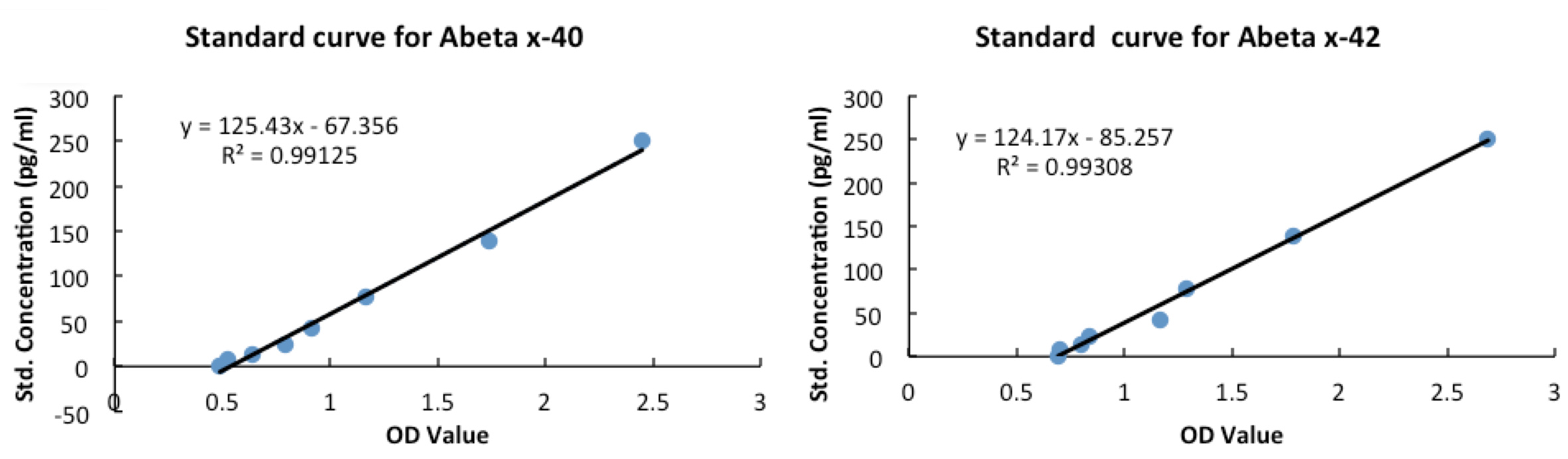


**Supplemental Figures**

**Supplemental Fig. 1.**

**
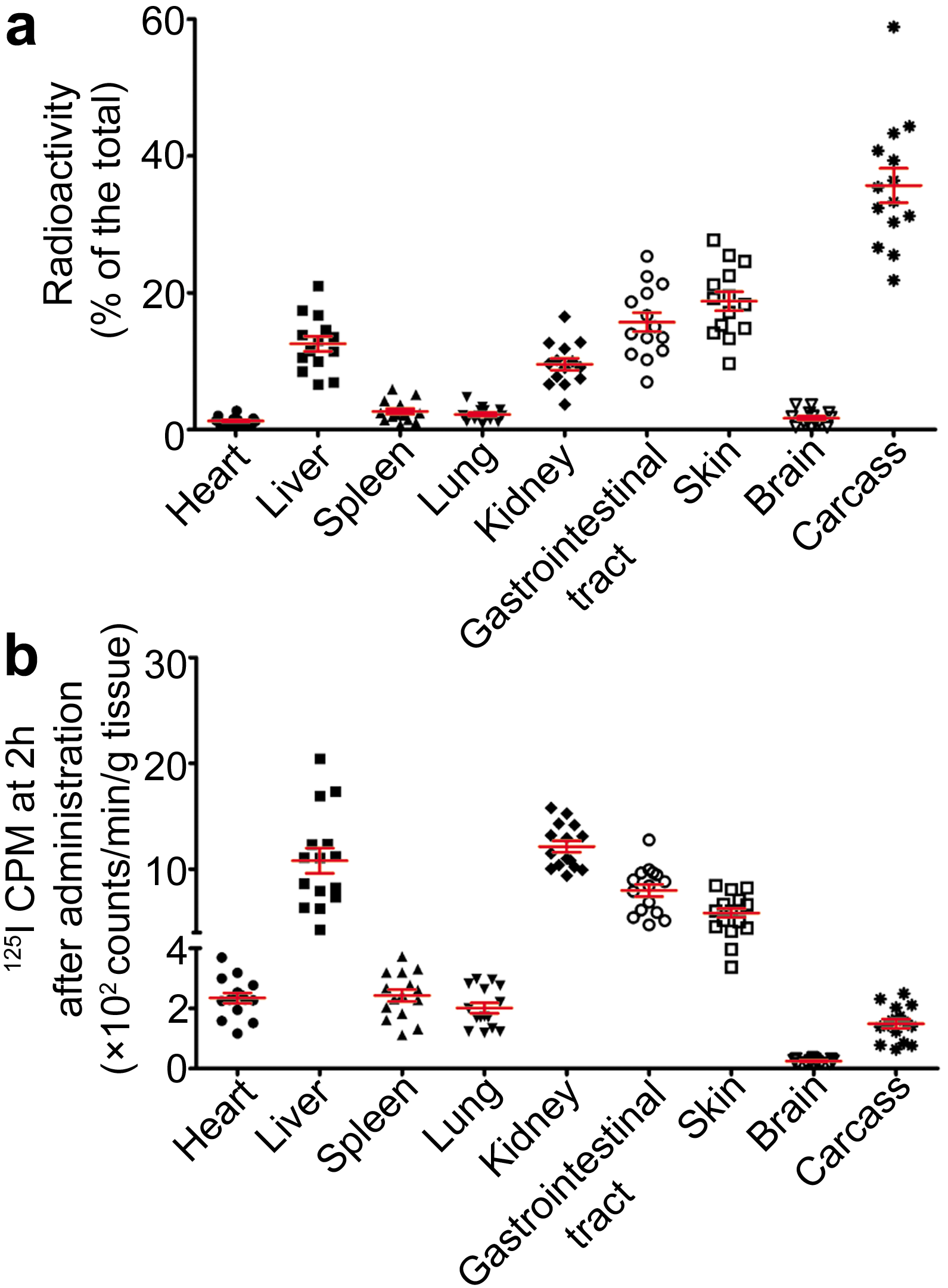
**

**Biodistribution of Aβ in peripheral organs and tissues.** A group of 3-month-old C57BL/6J mice (n=14) were intravenous bolus injected with 6.9MBq of the radiolabeled Aβ1-40 peptides. Two hours after injection, animals were euthanized, and the different samples of organs and tissues were collected, weighted and scanned for ^125^I radioactive intensity (CPM values). The values are expressed as % of total radioactivity (**a**) and counts/min/gram of tissue (**b**), respectively. Mean ± s.e.m..

**Supplemental Fig. 2.**


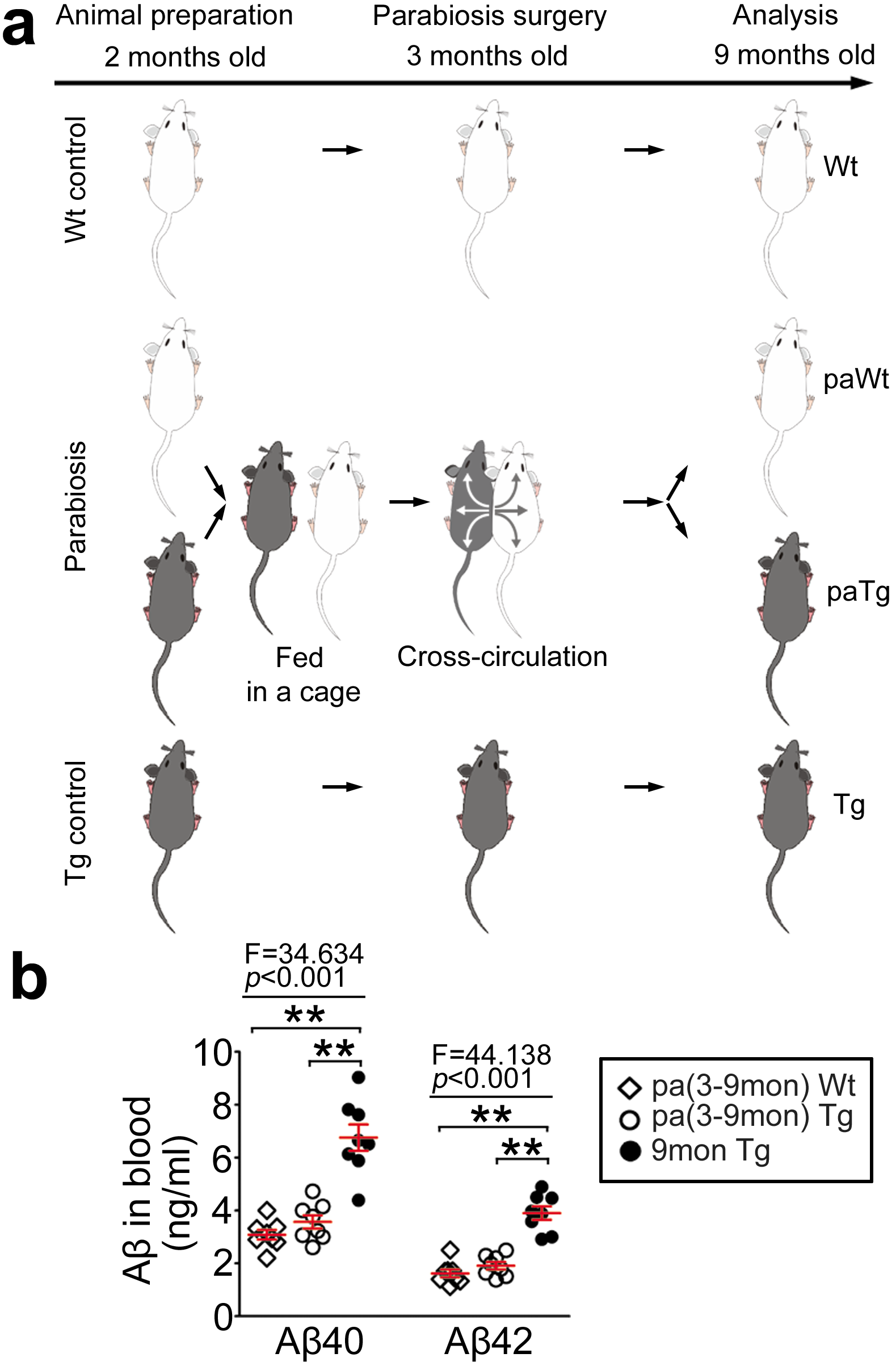


**Parabiosis between APPswe/PS1dE9 mice and their wild type littermates. (a)** Schematic depicting the parabiotic parings of the parabiosis from 3-months of age to 9-months of age. Female APPswe/PS1dE9 (Tg) mice aged 3 months (n=8) and their age-matched female wild type (Wt) littermates (n=8) were used for parabiosis. Parabiosis was performed before Aβ deposition at 3 months of age and samples were collected for analysis after Aβ deposition at 9 months of age. The age-matched female Wt and Tg mice without parabiosis (n=8) were used in parallel as controls. **(b)** Comparison of serum Aβ levels among parabiotic Tg mice, control Tg mice, and parabiotic Wt mice. n=8 per group, mean ± s.e.m., one-way ANOVA and Tukey’s test, **P*<0.05, ***P*<0.01.**Supplemental Fig. 3.**


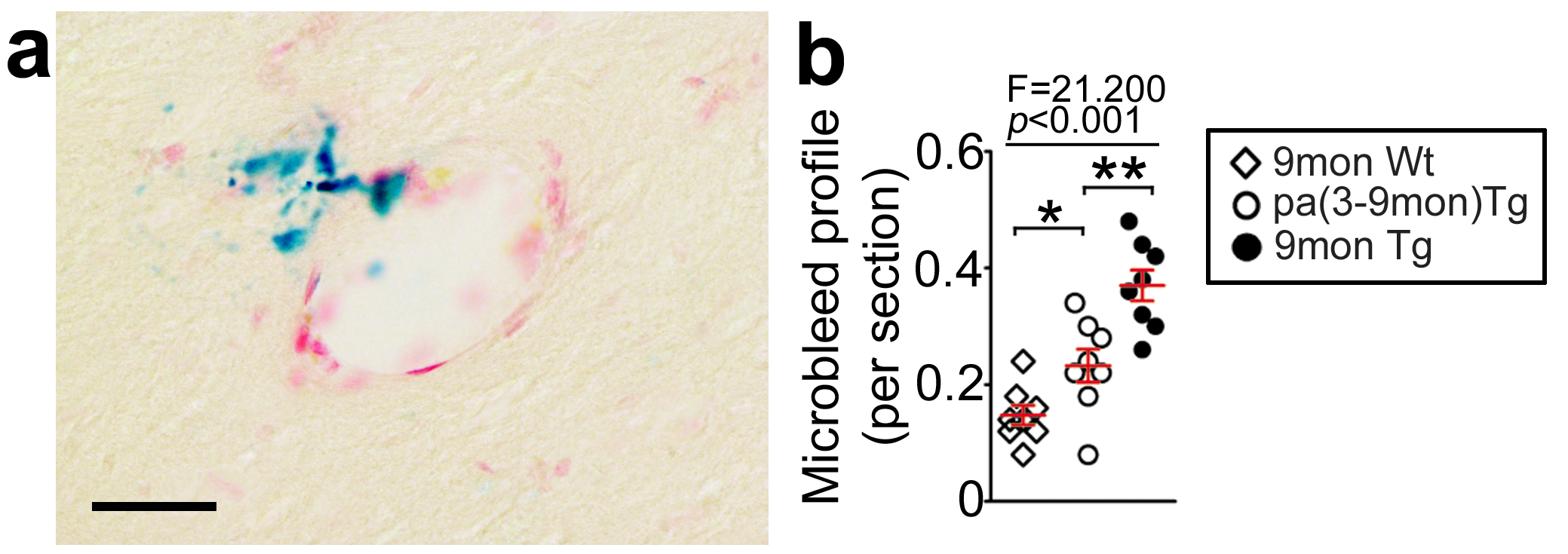


**Parabiosis reduces microbleed profiles in the brain of pa(3-9mon)Tg mice. (a)** Microbleed profiles were visualized using Prussian blue staining. Scale bars: 50μm. **(b)** Comparison of microbleed profile among pa(3-9mon)Tg mice, control Tg mice, and Wt mice. n = 8 per group, mean ± s.e.m., one-way ANOVA and Tukey’s test. **P*< 0.05, ***P*< 0.01. N.S. denotes no statistical significance.

**Supplemental Fig. 4.**

**
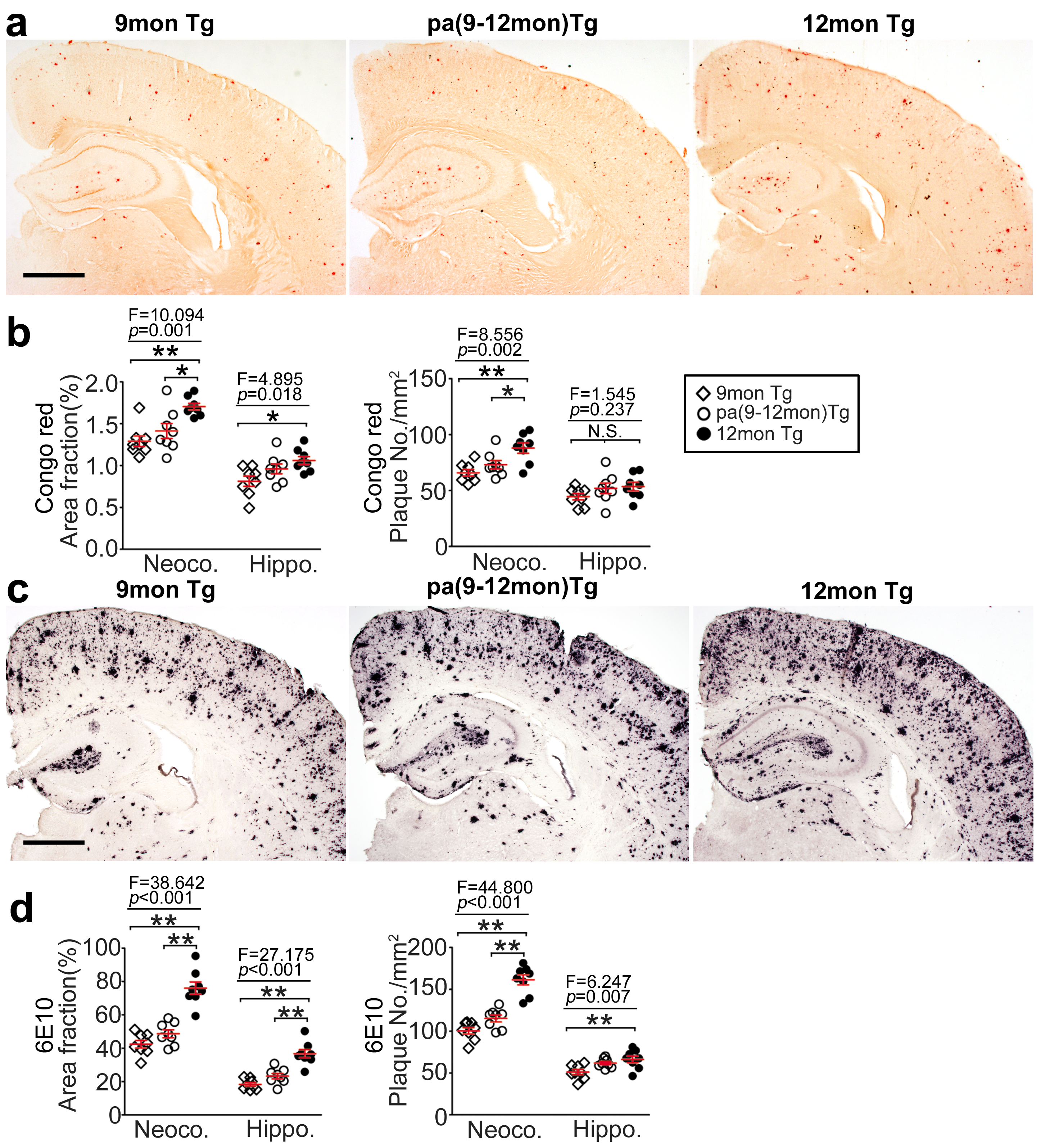
**

**Parabiosis reduces Aβ plaques in the brain of pa(9-12mon)Tg mice. (a)** Representative images of Congo red staining (representing compact Aβ plaques) in neocortex and hippocampus in 9mon Tg mice, pa(9-12mon) Tg mice, and 12mon Tg mice. Scale bars: 500μm. **(b)** Comparison of the area fraction and density of Congo red positive Aβ plaques in the neocortex (Neoco.) and hippocampus (Hippo.) among 9mon Tg mice, pa(9-12mon)Tg mice, and 12mon Tg mice. **(c)** Representative images of 6E10 immunohistochemical staining (representing total Aβ plaques) in neocortex and hippocampus in 9mon Tg mice, pa(9-12mon) Tg mice, and 12mon Tg mice. Scale bars: 500μm. **(d)** Comparison of the area fraction and density of 6E10-positive Aβ plaques in the neocortex (Neoco.) and hippocampus (Hippo.) among 9mon Tg mice, pa(9-12mon)Tg mice, and 12mon Tg mice. n = 8 per group, mean ± s.e.m.. one-way ANOVA and Tukey’s test.. **P*< 0.05, ***P*< 0.01. N.S. denotes no statistical significance.

**Supplemental Fig. 5.**


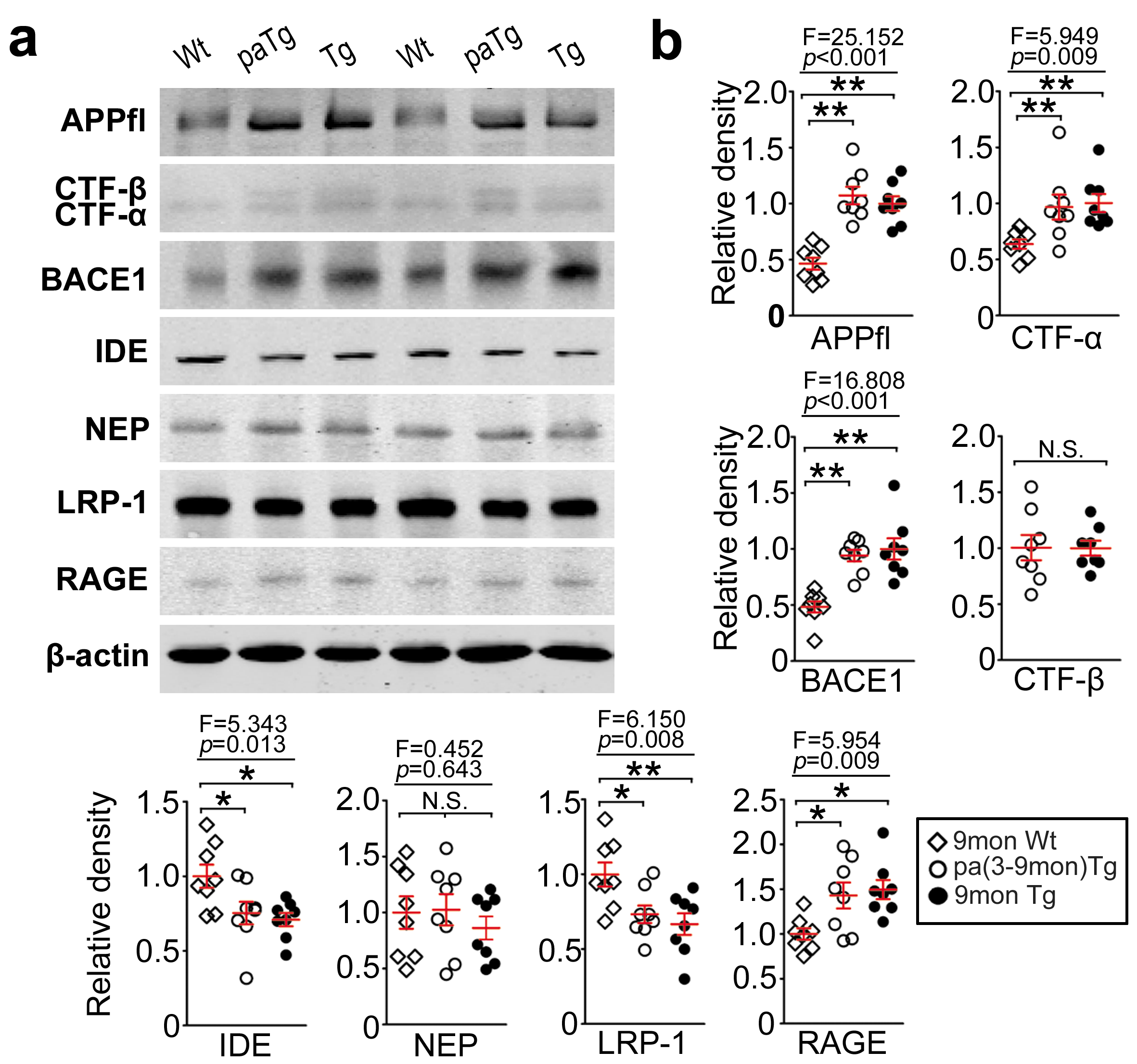


**Parabiosis does not change Aβ production, Aβ-degrading enzymes and receptors mediating Aβ transport across BBB in the brain of pa(3-9mon)Tg mice.** **(a)** Western blot assays of full length APP (APPfl) and its metabolites C-terminal fragment (CTF)-α and CTF-β, beta-secretase (BACE) -1, Aβ-degrading enzymes **[**insulin-degrading enzyme (IDE) and neprilysin (NEP)], receptors mediating Aβ transport across BBB [lipoprotein receptor-related protein (LRP)-1 and receptor for advanced glycation end-products (RAGE)] in brain homogenates of pa(3-9mon)Tg mice, control Tg mice, and Wt mice. **(b)** Comparisons of APPfl, CTF-α, CTF-β, BACE1, IDE, NEP, LRP-1, and RAGE in brain homogenates among pa(3-9mon)Tg mice, control Tg mice, and Wt mice. n = 8 per group, mean ± s.e.m.. 2-tailed *t* – test for CTF-β. One-way ANOVA and Tukey’s test for the rest. **P*< 0.05, ***P*< 0.01. N.S. denotes no statistical significance.

**Supplemental Fig. 6.**


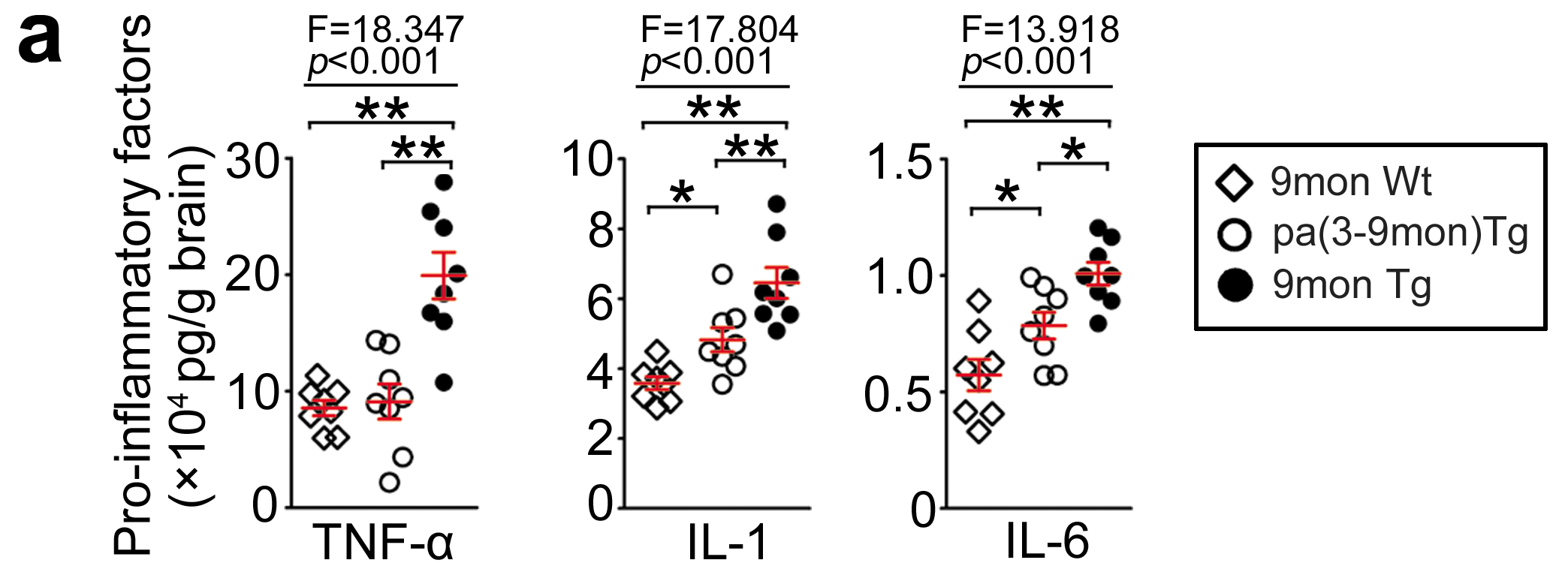


**Parabiosis reduces levels of proinflammatory cytokines in the brain of pa(3-9mon)Tg mice. (a)** Comparison of TNF-α, IL-1, and IL-6 in the brain homogenates among pa(3-9mon)Tg mice, control Tg mice, and Wt mice. n = 8 per group, mean ± s.e.m., one-way ANOVA and Tukey’s test. **P*< 0.05, ***P*< 0.01.
